# What do Cochrane systematic reviews say about interventions for autism spectrum disorders? O que as revisões sistemáticas Cochrane falam sobre intervenções para os transtornos do espectro autista?

**DOI:** 10.1590/1516-3180.2017.0058200317

**Published:** 2017-04-20

**Authors:** Larissa Lyra, Luiz Eduardo Rizzo, Camila Sá Sunahara, Daniela Vianna Pachito, Carolina de Oliveira Cruz Latorraca, Ana Luiza Cabrera Martimbianco, Rachel Riera

**Affiliations:** I Undergraduate Medical Student, Escola Paulista de Medicina (EPM), Universidade Federal de São Paulo (Unifesp), São Paulo (SP), Brazil.; II MD, MSc. Neurologist; Postgraduate Student, Evidence-Based Health Program, Universidade Federal de São Paulo (Unifesp), São Paulo (SP), Brazil; and Assistant Researcher at Cochrane Brazil, São Paulo (SP), Brazil.; III Psychologist. Postgraduate Student, Evidence-Based Health Program, Universidade Federal de São Paulo (Unifesp), São Paulo (SP), Brazil; and Assistant Researcher, Cochrane Brazil, São Paulo (SP), Brazil.; IV MSc, PhD. Physiotherapist and Assistant Researcher, Cochrane Brazil, São Paulo (SP), Brazil.; V MD, MSc, PhD. Rheumatologist and Adjunct Professor, Discipline of Evidence-Based Medicine, Escola Paulista de Medicina (EPM), Universidade Federal de São Paulo (Unifesp); and Assistant Coordinator, Cochrane Brazil, São Paulo (SP), Brazil.

**Keywords:** Autism spectrum disorder, Therapeutics, Review [Publication type], Evidence-based practice, Evidence-based medicine

## Abstract

**CONTEXT AND OBJECTIVE:**

Autism spectrum disorders (ASDs) include autistic disorder, Asperger’s disorder and pervasive developmental disorder. The manifestations of ASDs can have an important impact on learning and social functioning that may persist during adulthood. The aim here was to summarize the evidence from Cochrane systematic reviews on interventions for ASDs.

**DESIGN AND SETTING:**

Review of systematic reviews, conducted within the Discipline of Evidence-Based Medicine, Escola Paulista de Medicina, Universidade Federal de São Paulo.

**METHODS:**

We included and summarized the results from Cochrane systematic reviews on interventions for ASDs.

**RESULTS:**

Seventeen reviews were included. These found weak evidence of benefits from acupuncture, gluten and casein-free diets, early intensive behavioral interventions, music therapy, parent-mediated early interventions, social skill groups, Theory of Mind cognitive model, aripiprazole, risperidone, tricyclic antidepressants and selective serotonin reuptake inhibitors (SSRI); this last only for adults. No benefits were found for sound therapies, chelating agents, hyperbaric oxygen therapy, omega-3, secretin, vitamin B6/magnesium and SSRI for children.

**CONCLUSION:**

Acupuncture, gluten and casein-free diets, early intensive behavioral interventions, music therapy, parent-mediated early interventions, social skill groups and the Theory of Mind cognitive model seem to have benefits for patients with autism spectrum disorders (very low to low-quality evidence). Aripiprazole, risperidone, tricyclic antidepressants and SSRI (this last only for adults) also showed some benefits, although associated with higher risk of adverse events. Experimental studies to confirm a link between probable therapies and the disease, and then high-quality long-term clinical trials, are needed.

## RESUMO

**CONTEXTO E OBJETIVO:**

Os transtornos do espectro autista (TEA) incluem autismo, doença de Asperger e transtorno global do desenvolvimento. As manifestações dos TEA podem ter importante impacto na aprendizagem e funcionamento social, que pode persistir durante a fase adulta. O objetivo foi resumir as evidências de revisões sistemáticas Cochrane sobre intervenções para TEA.

**TIPO DE ESTUDO E LOCAL:**

Revisão de revisões sistemáticas conduzida na Disciplina de Medicina Baseada em Evidências da Escola Paulista de Medicina, Universidade Federal de São Paulo.

**MÉTODOS:**

Nós incluímos e resumimos os resultados de revisões sistemáticas Cochrane sobre intervenções para TEA.

**RESULTADOS:**

Foram incluídas 17 revisões que encontraram evidências fracas de benefícios da acupuntura, dietas isentas de glúten e caseína, intervenção comportamental intensiva precoce, musicoterapia, intervenção precoce mediada pelos pais, grupos de habilidades sociais, modelo cognitivo de Teoria da Mente, aripiprazol, risperidona, antidepressivos tricíclicos, inibidores seletivos da recaptação da serotonina (ISRS); o último apenas para adultos. Nenhum benefício foi encontrado com terapias sonoras, agentes quelantes, oxigenoterapia hiperbárica, ômega-3, secretina, vitamina B6/magnésio e ISRS para crianças.

**CONCLUSÃO:**

Acupuntura, dietas sem glúten e caseína, intervenção comportamental intensiva precoce, musicoterapia, intervenção precoce mediada pelos pais, grupos de habilidades sociais e modelo cognitivo de Teoria da Mente parecem ter benefícios para pacientes com TEA (evidência de qualidade muito baixa a baixa). Aripiprazol, risperidona, antidepressivos tricíclicos e ISRS (o último apenas para adultos) também apresentam algum benefício, embora estejam associados a maior risco de eventos adversos. Estudos experimentais confirmando a relação entre prováveis terapias e a doença, e então ensaios clínicos de alta qualidade e de longo seguimento, são necessários.

## INTRODUCTION

Autism spectrum disorders (ASDs) have an estimated prevalence ranging from 3.3 to 116 children per 10,000 and it is widely accepted that autism affects approximately 1% of children worldwide.^1^ This may reflect increased access to diagnosis, concern among healthcare professionals and parents, more sensitive diagnostic criteria and/or a true increase in prevalence.^2^

ASDs include autistic disorder, Asperger’s disorder and pervasive developmental disorder. Patients with these conditions present deficits in communication, social interaction and cognitive function; problems with feeding; hypo or hypersensitivity; and, sometimes, self-harmful behavior.^3^ With greater severity of ASDs, activities of daily living are limited and the impairments of ASDs can have an important impact on learning and social functioning that may persist during adulthood.^4^

Healthcare and social care for people with ASDs is frequently complex, since they are more likely to have mental health comorbidities and suicidal ideation.^5^ Assessments need to be multidisciplinary and developmental, and early detection is determinant. Therapeutic interventions need to be personalized, focusing on the specific clinical features presented by each patient.^6^

Individual or parent-based psychosocial interventions are frequently used in clinical practice, with the aim of improving clinical features relating to communication, cognition, behavior and relationships, for instance. Pharmacological therapy is an option for mental health comorbidities. New therapeutic approaches include genetic and pharmacological strategies for reducing synthesis of specific proteins and inhibiting pleiotropic growth factors.^7^ Concerning the time for starting the treatment, randomized controlled trials have added new evidence that, for many children aged up to three years, early intervention can improve outcomes, thus increasing the potential benefits of early diagnosis facilitated by early screening.^8^^,^^9^

Despite recent advances in this area, the evidence regarding autism remains limited and this condition continues to be a challenge for researchers, healthcare professionals, patients, parents and caregivers.

## OBJECTIVES

To map out and summarize all Cochrane systematic reviews on interventions for autism spectrum disorders, and present the results on the basis of the quality of the evidence.

## METHODS

### Design

Review of Cochrane systematic reviews on interventions for ASD.

### Setting

Discipline of Evidence-Based Medicine, Escola Paulista de Medicina, Universidade Federal de São Paulo (UNIFESP), Brazil.

### Criteria for including reviews

#### Types of study

We included completed Cochrane systematic reviews, with no restriction regarding date of publication. Protocols for systematic reviews and reviews signaled as “withdrawn” in the Cochrane Database of Systematic Reviews (CDSR) were not considered. We included only the latest version of each review.

#### Types of participants

Individuals with ASD.

#### Types of interventions

All types of interventions (pharmacological and non-pharmacological) aiming to treat ASD.

#### Type of outcomes

Clinical and laboratory outcomes were considered, as presented by the systematic reviews.

### Searching for reviews

We conducted systematic searches in the Cochrane Database of Systematic Reviews (CDSR) (via Wiley) using a sensitive search strategy (Table 1).

**Table 1:**
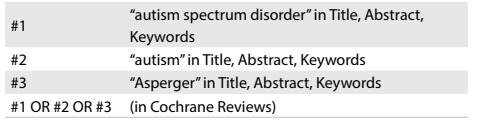
Search strategy and results from Cochrane Database of Systematic Reviews (conducted on November 22, 2016)

### Selecting reviews

Three reviewers evaluated the titles and abstracts of records that had initially been retrieved on the basis of the inclusion criteria. The full texts of records with the potential for inclusion were read to confirm whether they should be included (reasons for exclusions were recorded and presented). Divergences between reviewers were resolved through reaching a consensus.

### Presenting the results

We used a narrative structure (qualitative synthesis) to present the results from the systematic reviews included.

## RESULTS

### Search results

The initial search retrieved 24 reviews. From these, we excluded six reviews addressing other clinical situations (X fragile syndrome, prematurity and attention deficit hyperactivity disorder) and one that was a previous version of a review that we included. Thus, we included 17 systematic reviews.^4^^,^^10^^,^^11^^,^^12^^,^^13^^,^^14^^,^^15^^,^^16^^,^^17^^,^^18^^,^^19^^,^^20^^,^^21^^,^^22^^,^^23^^,^^24^^,^^25^

### Results from systematic reviews

A summary of each systematic review is presented narratively below. In addition, Table 2 ^4^^,^^10^^,^^11^^,^^12^^,^^13^^,^^14^^,^^15^^,^^16^^,^^17^^,^^18^^,^^19^^,^^20^^,^^21^^,^^22^^,^^23^^,^^24^^,^^25^ presents the issues addressed, the main findings from each systematic review and the quality of the evidence (based on the GRADE approach).^26^

**Table 2:**
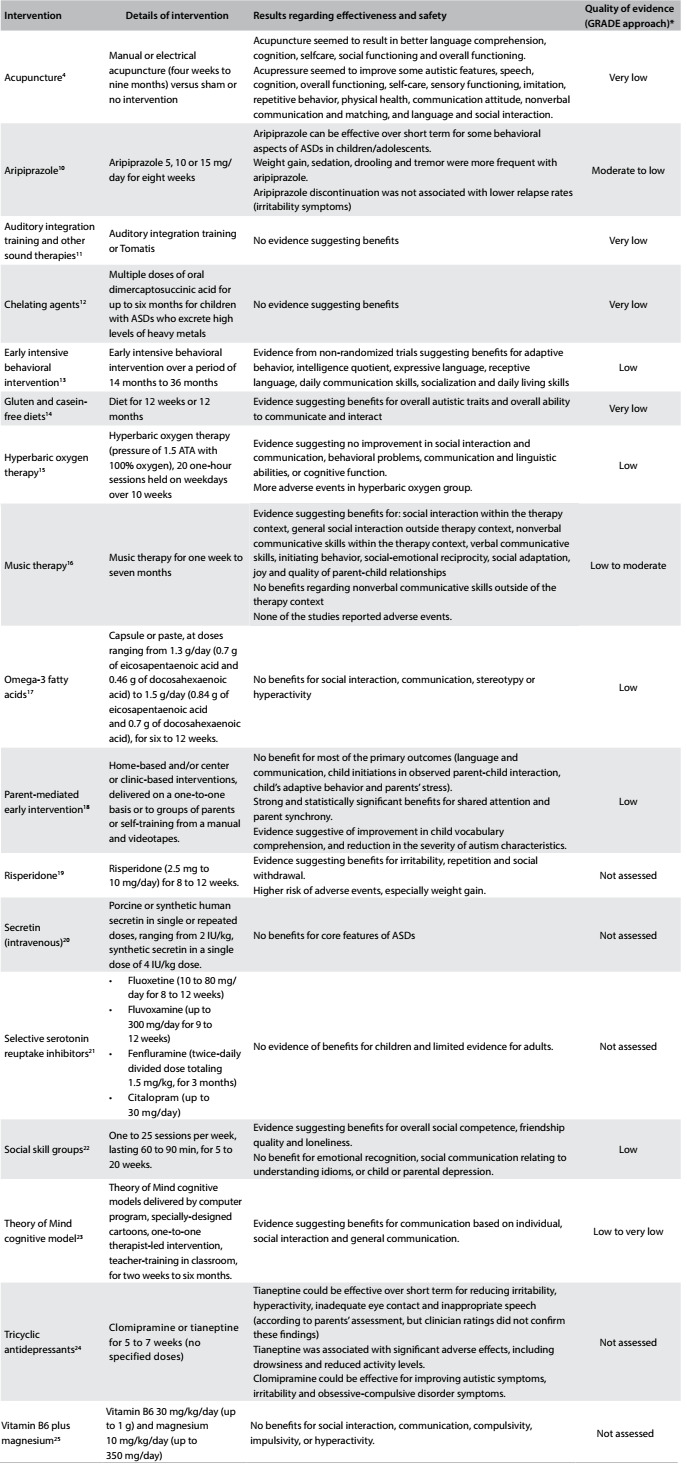
Characteristics and main results from systematic reviews included

ASD = autism spectrum disorders; *GRADE = Grading of Recommendations Assessment, Development and Evaluation. Tool used for assessing the quality of the body of the current evidence. High quality: low probability that further studies will change the confidence regarding the existing evidence. Moderate quality: some probability that further studies will change the confidence regarding the existing evidence. Low quality: high probability that further studies will change the confidence regarding the existing evidence. Very low quality: there is much uncertainty about the information, precluding any valid interpretation.^26^

#### 1. Acupuncture

The review^4^ considered randomized controlled trials (RCTs) and quasi-randomized controlled trials and included 10 studies (from low to high methodological quality) on 390 children aged from 3 to 18 years who presented ASDs and received acupuncture for periods ranging from four weeks to nine months. The following results were found:

- Needle acupuncture (by means of manual or electrical stimulation) versus sham: no difference in core autistic features. On the Ritvo-Freeman Real Life Rating Scale (RFRLRS): mean difference (MD) = 0.09; 95% confidence interval (CI) = -0.03 to 0.21; P = 0.16. Acupuncture seemed to result in better language comprehension, cognition, selfcare and social functioning, and a higher probability of improvement in overall functioning greater than or equal to 25%.
- Needle acupuncture (by means of manual or electrical stimulation) versus no treatment: acupuncture seemed to improve autistic features, speech, cognition, overall functioning, self-care, sensory functioning, imitation, repetitive behavior and physical health.
- Acupressure versus no treatment: acupressure might result in improvement in overall functioning, communication attitude, non-verbal communication and matching, and in language and social interaction.

Most of the favorable outcomes were observed only in a single study and few outcomes were supported by pooled results from more than one study. The majority of the effect size estimates for the significant outcomes had imprecisions with a wide confidence interval. These limitations mean that the significant result was not sufficiently robust to enable a reliable conclusion. 

Therefore, the authors concluded that the current evidence did not support use of acupuncture for treating ASDs. For further details, the original abstract can be consulted, available from: http://onlinelibrary.wiley.com/doi/10.1002/14651858.CD007849.pub2/abstract.

#### 2. Aripiprazole

Antipsychotics have been used as medications for ASD-related irritability. The review^10^ included three RCTs on children and adolescents (aged from 9 to 17 years): two were short-term studies (eight weeks) on the effects of aripiprazole on behavioral problems among 316 children/adolescents; one was a longer-term study (up to 16 weeks) in which 85 children/adolescents whose symptoms initially improved through aripiprazole were withdrawn from the medication to assess whether their behavioral problems would recur. All three RCTs were supported by Bristol-Myers Squibb (Princeton, NJ, United States) and Otsuka Pharmaceutical Company, Ltd. (Tokyo, Japan), and had editorial support from Ogilvy Healthworld Medical Education and Bristol-Myers Squibb. The results from a meta-analysis showed that there was an improvement favoring aripiprazole, in comparison with placebo, for the following outcomes:

- Aberrant Behavior Checklist (ABC) - irritability subscale (MD = -6.17 points; 95% CI = -9.07 to -3.26; two RCTs; 308 participants; moderate-quality evidence);
- ABC hyperactivity subscale (MD = -7.93 points; 95% CI = -10.98 to -4.88; two RCTs; 308 participants, moderate-quality evidence);
- ABC stereotypy subscale (MD = -2.66 points; 95% CI = -3.55 to -1.77; two RCTs; 308 participants, moderate-quality evidence).

In terms of side effects, aripiprazole was associated with greater increase in weight (MD = 1.13 kg; 95% CI = 0.71 to 1.54; two RCTs; 308 participants; moderate-quality evidence), higher risk of sedation (risk ratio [RR] = 4.28; 95% CI = 1.58 to 11.60; two RCTs; 313 participants; moderate-quality evidence) and tremor (RR = 10.26; 95% CI = 1.37 to 76.63; two RCTs; 313 participants; moderate-quality evidence). The discontinuation RCT study showed no difference in relapse rate, with regard to symptoms of irritability (hazard ratio [HR] = 0.57; 95% CI = 0.28 to 1.12; 85 participants; low-quality evidence). 

The authors concluded that aripiprazole might be effective as a short-term intervention for some behavioral aspects of ASDs among children/adolescents. However, they stated that notable side effects, including weight gain, sedation, drooling and tremor, needed to be considered. For further details, the original abstract can be consulted, available from: http://onlinelibrary.wiley.com/doi/10.1002/14651858.CD009043.pub3/abstract.

#### 3. Auditory integration training and other sound therapies

Auditory integration therapies (including Tomatis therapy and Samonas sound therapy) are techniques for improving abnormal sound sensitivity in individuals with behavioral disorders including ASDs. The review^11^ identified six RCTs that used auditory integration therapy and one that used Tomatis therapy (182 participants aged three to 39 years). Meta-analysis was not possible due to the huge heterogeneity among the RCTs or the reports of data in unusable forms. Three RCTs did not show any benefit from auditory integration therapy over control interventions. Three RCTs reported improvements from auditory integration therapy on the Aberrant Behavior Checklist, after three months, but they used a total score rather than subgroup scores, which is questionable. The study addressing Tomatis therapy did not find any difference between the treatment and control conditions regarding language.

The authors concluded that, considering the methodological limitations of the existing RCTs, no evidence supporting the use of auditory integration therapy or other sound therapies was available up to that time. For further details, the original abstract can be consulted, available from: http://onlinelibrary.wiley.com/doi/10.1002/14651858.CD003681.pub3/abstract.

#### 4. Chelating agents for autism spectrum disorders (ASDs)

It has been suggested that the severity of autism spectrum disorder (ASD) symptoms is associated with the levels of serum or stored toxic metals, and that chelating agents improve symptoms. The review^12^ aimed to assess the effects of these agents on ASDs and included one randomized clinical trial (RCT) with 49 children. This RCT compared multiple doses of oral dimercaptosuccinic acid (DMSA), for up to six months versus placebo among children who excreted high levels of heavy metals.

The authors concluded that there was no evidence to suggest that multiple rounds of oral DMSA had any effect on ASD symptoms. Considering previous reports of serious adverse events, including hypocalcemia, renal impairment and reported death, they stated that the risks involved in chelation to treat ASDs currently outweighed the benefits. Evidence supporting a causal relation between heavy metals and autism would be needed before further trials were conducted. For further details, the original abstract can be consulted, available from: http://onlinelibrary.wiley.com/doi/10.1002/14651858.CD010766.pub2/abstract.

#### 5. Early intensive behavioral intervention

Early intensive behavioral intervention (EIBI) is based on the principles of applied behavioral analysis on interventions delivered over many years for 20 to 40 hours per week. For the review,^13^ the participants needed to have been less than six years of age at treatment onset and to have been assigned to their study condition prior to starting treatment. This review included one RCT and four non-randomized trials (203 participants) that used a treatment-as-usual comparison group. Positive effects favoring EIBI were found for all the following outcomes:

- Adaptive behavior (standardized mean difference using Hedges g = 0.69; 95% CI = 0.38 to 1.01; P < 0.0001);
- Intelligence quotient, IQ (g = 0.76; 95% CI = 0.40 to 1.11; P < 0.0001);
- Expressive language (g = 0.50; 95% CI = 0.05 to 0.95; P = 0.03);
- Receptive language (g = 0.57; 95% CI = 0.20 to 0.94; P = 0.03);
- Daily communication skills (g = 0.74; 95% CI = 0.30 to 1.18; P = 0.0009);
- Socialization (g = 0.42; 95% CI = 0.11 to 0.73; P = 0.0008);
- Daily living skills (g = 0.55; 95% CI = 0.24 to 0.87; P = 0.0005).

The authors concluded that there was some evidence that EIBI was effective for children with ASDs. However, they considered that the current state of the evidence was limited because the data came from non-randomized studies. For further details, the original abstract can be consulted, available from: http://onlinelibrary.wiley.com/doi/10.1002/14651858.CD009260.pub2/abstract.

#### 6. Gluten and casein-free diets 

It has been hypothesized that peptides from gluten and casein may participate in the origins of autism and that that physiology and psychology of autism might be explained in terms of excessive opioid activity linked to these peptides. The review^14^ included two small RCTs (35 participants) assessing the effects of gluten and casein-free diets for ASDs. No meta-analysis was possible, due to different outcome measurements. There were significant treatment effects favoring the dietary intervention for overall autistic traits (MD = -5.60; 95% CI = -9.02 to -2.18; P = 0.001) and the overall ability to communicate and interact (MD = 1.70; 95% CI = 0.50 to 2.90). No adverse events were reported.

The authors concluded that, despite the high rates of use of gluten and/or casein-free diets for children with ASDs, the current evidence was not robust enough to support these practices. For further details, the original abstract can be consulted, available from: http://onlinelibrary.wiley.com/doi/10.1002/14651858.CD003498.pub3/abstract.

#### 7. Hyperbaric oxygen therapy

It has been hypothesized that hyperbaric oxygen therapy might reduce the biochemical dysfunction and clinical symptoms of ASDs. The review^15^ included one RCT (60 children) comparing hyperbaric oxygen therapy and sham treatment, and the results showed that there were no improvements in social interaction and communication, behavioral problems, communication and linguistic abilities, or cognitive function. There were more adverse events (odds ratio [OR)] = 3.87; 95% CI = 1.53 to 9.82) and more children who experienced adverse events (OR = 4.40; 95% CI = 1.33 to 14.48) in the hyperbaric oxygen group.

The authors concluded that up to that time, there was no evidence that hyperbaric oxygen therapy improved the core symptoms and associated symptoms of ASDs. They stated that it was important to consider that adverse events could occur. For further details, the original abstract can be consulted, available from: http://onlinelibrary.wiley.com/doi/10.1002/14651858.CD010922.pub2/abstract.

#### 8. Music therapy

The review^16^ is an updated version of a review published in 2006 that aimed to assess the effects of music therapy for ASD. Ten RCTs (165 participants) examined the short and medium-term effects of music therapy over periods of one week to seven months for children with ASDs. Music therapy was better than “placebo” therapy or the usual care for the following outcomes:

- Social interaction within the therapy context (standard mean difference [SMD] = 1.06; 95% CI = 0.02 to 2.10; one RCT; 10 participants);
- General social interaction outside of therapy context (SMD = 0.71; 95% CI = 0.18 to 1.25; three RCTs; 57 participants; moderate-quality evidence);
- Nonverbal communicative skills within the therapy context (SMD = 0.57; 95% CI = 0.29 to 0.85; three RCTs; 30 participants);
- Verbal communicative skills (SMD = 0.33; 95% CI = 0.16 to 0.49; six RCTs; 139 participants);
- Initiating behavior (SMD = 0.73; 95% CI = 0.36 to 1.11; three RCTs; 22 participants; moderate-quality evidence);
- Social-emotional reciprocity (SMD = 2.28; 95% CI = 0.73 to 3.83; one RCT; 10 participants; low-quality evidence);
- Social adaptation (SMD = 0.41; 95% CI = 0.21 to 0.60; four RCTs; 26 participants);
- Joy (SMD = 0.96; 95% CI = 0.04 to 1.88; one RCT; 10 participants); and
- Quality of parent-child relationships (SMD = 0.82; 95% CI = 0.13 to 1.52; two RCTs; 33 participants; moderate-quality evidence).

There was no difference in nonverbal communicative skills outside of the therapy context (SMD = 0.48; 95% CI = -0.02 to 0.98; three RCTs; 57 participants; low-quality evidence). None of the studies reported any adverse events. For further details, the original abstract can be consulted, available from: http://onlinelibrary.wiley.com/doi/10.1002/14651858.CD004381.pub3/abstract.

#### 9. Omega-3 fatty acid supplementation

The review^17^ included two RCTs (37 children) comparing omega-3 fatty acid supplementation versus placebo. No evidence was found that omega-3 supplements had any effect on social interaction (MD = 0.82; 95% CI = -2.84 to 4.48), communication (MD = 0.62; 95% CI = -0.89 to 2.14), stereotypy (MD = 0.77; 95% CI = -0.69 to 2.22) or hyperactivity (MD = 3.46, 95% CI = -0.79 to 7.70). 

The authors concluded that, up to that time, there was no high-quality evidence that omega-3 fatty acid supplementation was effective for children with ASDs. For further details, the original abstract can be consulted, available from: http://onlinelibrary.wiley.com/doi/10.1002/14651858.CD007992.pub2/abstract.

#### 10. Parent-mediated early intervention

Approaches that help parents develop strategies for interactions and management of behavior can be useful in cases of ASDs. The review^18^ included 17 RCTs on 919 children with ASDs. The following results were found:

- No benefit for most of the primary outcomes (language and communication, child initiations in observed parent-child interaction, child’s adaptive behavior and parents’ stress);
- Strong and statistically significant benefits for patterns of parent-child interaction: shared attention (SMD = 0.41; 95% CI = 0.14 to 0.68) and parent synchrony (SMD = 0.90; 95% CI = 0.56 to 1.23);
- Evidence suggestive of improvement of child vocabulary comprehension reported by parents (MD = 36.26; 95% CI = 1.31 to 71.20) and reduction in the severity of children’s autism characteristics (SMD = -0.30; 95% CI = -0.52 to -0.08).

For further details, the original abstract can be consulted, available from: http://onlinelibrary.wiley.com/doi/10.1002/14651858.CD009774.pub2/abstract.

#### 11. Risperidone

The review^19^ included three RCTs (211 adults or children) comparing risperidone (2.5 mg to 10 mg/day) with placebo over periods of eight to 12 weeks. Some benefits were found regarding irritability, repetition and social withdrawal. However, these needed to be considered in relation to the adverse events, especially weight gain.

The authors concluded that risperidone might be beneficial in relation to some features of autism, but that the evidence was limited because the existing studies had small sample sizes with short follow-ups, and because of the lack of a single standardized outcome measurement. For further details, the original abstract can be consulted, available from: http://onlinelibrary.wiley.com/doi/10.1002/14651858.CD005040.pub2/abstract.

#### 12. Secretin

Secretin, a gastrointestinal hormone, has been suggested as an effective treatment for ASDs based on anecdotal evidence. The review^20^ included 16 heterogeneous RCTs (two of these were conducted by Repligen, a pharmaceutical company) in which over 900 children were recruited to receive intravenous secretin (porcine or synthetic, in single or multiple doses) or placebo. Twenty-five established standardized outcome measurements assessing communication, behavior, visuospatial skills, effects and adverse events were reported.

The authors concluded that there was no evidence that secretin was effective and that therefore currently it could not be recommended and should not be administered as a treatment for ASDs. They stated that further RCTs would only be justifiable if there was any new high-quality replicable scientific evidence proving a link between secretin and ASDs. For further details, the original abstract can be consulted, available from: http://onlinelibrary.wiley.com/doi/10.1002/14651858.CD003495.pub3/abstract.

#### 13. Selective serotonin reuptake inhibitors (SSRIs)

The review^21^ included nine heterogeneous RCTs (320 adults or children) assessing fluoxetine (three RCTs), fluvoxamine (two RCTs), fenfluramine (two RCTs) and citalopram (two RCTs). Varying inclusion criteria were used with regard to diagnostic criteria and the participants’ intelligence quotient. Due to heterogeneity, the data were unsuitable for meta-analysis, except for one outcome (proportional improvement). The results did not show any evidence of positive effects from citalopram for children with ASDs (one high-quality RCT). Three small RCTs on adults showed positive outcomes for clinical global impression (CGI) and obsessive-compulsive behavior (OCB); one RCT showed improvements in aggression; and another in relation to anxiety.

The authors concluded that there was no evidence of effects from SSRIs on children and that the evidence relating to adults was limited due to the small size of the studies, with unclear risk of bias. For further details, the original abstract can be consulted, available from: http://onlinelibrary.wiley.com/doi/10.1002/14651858.CD004677.pub3/abstract.

#### 14. Social skill groups

Major difficulties in social interactions have been found to be a defining feature of individuals with ASDs. The review^22^ included five RCTs (196 participants aged 6 to 21 years). The results showed that social skill groups improved overall social competence (SMD = 0.47; 95% CI = 0.16 to 0.78; P = 0.003), friendship quality (SMD = 0.41; 95% CI = 0.02 to 0.81; P = 0.04) and loneliness (SMD = -0.66; 95% CI -1.15 to -0.17; one RCT). No differences were found regarding emotional recognition (SMD = 0.34; 95% CI = -0.20 to 0.88; two RCTs), social communication in relation to understanding idioms (SMD = 0.05; 95% CI = -0.63 to 0.72; one RCT) or child or parental depression. No adverse events were reported. Given the nature of the intervention and the outcome measurements selected, the risks of performance and detection bias were high. There was only limited external validity for these results, since all the RCTs were conducted in the United States, focusing mainly on children aged 7 to 12, and the participants were all of average or above average intelligence.

The authors concluded that there was some evidence that social skill groups might improve social competence for some children and adolescents with ASDs. They stated that further research was needed in order to reach conclusions that would be more robust, especially with regard to improvements in quality of life. For further details, the original abstract can be consulted, available from: http://onlinelibrary.wiley.com/doi/10.1002/14651858.CD008511.pub2/abstract.

#### 15. Theory of Mind cognitive model

The Theory of Mind (ToM) model suggests that people with ASDs have difficulty in understanding the minds (emotions, feelings, beliefs and thoughts) of other people. Interventions to teach ToM for patients could provide some benefits for ASDs. The review^23^ included 22 heterogeneous RCTs (695 participants). Evidence of some benefit was found for communication based on individual results (three RCTs; very low-quality evidence), social interaction (11 RCTs; low-quality evidence), general communication (4 RCTs; very low-quality evidence) and ToM ability (4 RCTs; very low-quality evidence). The meta-analysis showed that interventions targeting recognition of emotions across age groups and working with people within the average range of intellectual ability had a positive effect on the target skill (through a test using photographs of faces; mean increase = 0.75 points; 95% CI = 0.22 to 1.29 points; four RCTs; 105 participants). It was found that therapist-led joint-attention approaches could improve joint attention behavior within adult-child interactions (mean increase = 0.55 points; 95% CI = 0.11 to 0.99 points; two RCTs; 88 participants). However, further analysis undermined this conclusion by demonstrating that there was no clear evidence that intervention could improve joint-attention initiations as measured using a standardized assessment tool (mean increase = 0.23 points, 95% CI = -0.48 to 0.94 points; three RCTs; 92 participants). No adverse effects were observed.

The authors concluded that there was some evidence that teaching ToM to people with ASD seemed to present benefits. However, inconsistencies in the findings and measurements meant that that the evidence was of very low or low quality, which reduced the confidence in these findings. For further details, the original abstract can be consulted, available from: http://onlinelibrary.wiley.com/doi/10.1002/14651858.CD008785.pub2/abstract.

#### 16. Tricyclic antidepressants

Because of the impact of tricyclic antidepressants (TCAs) on serotonins, they have been used to treat ASDs. The review^24^ included three RCTs assessing clomipramine (two RCTs) or tianeptine (one RCT), for children and young adults with ASDs. Due to heterogeneity among the study participants, types of TCAs and outcomes measured, no meta-analysis was performed. One study showed that tianeptine could be effective over the short term for reducing irritability, hyperactivity, inadequate eye contact and inappropriate speech, but clinician ratings found that it did not have any significant impact on these symptoms. There were also significant adverse effects, including increased drowsiness and reduced activity levels. For clomipramine, there was evidence of improvement in autistic symptoms, irritability and obsessive-compulsive disorder symptoms. However, there was conflicting evidence in relation to hyperactivity across the two studies on clomipramine, and no significant changes were found regarding inappropriate speech. There were significant dropout rates in the clomipramine arm of one study.

The authors concluded that since there was limited and conflicting evidence regarding the effects and side effects of TCAs for this purpose, further research would be required before TCAs could be recommended for ASDs. For further details, the original abstract can be consulted, available from: http://onlinelibrary.wiley.com/doi/10.1002/14651858.CD008372.pub2/abstract.

#### 17. Vitamin B6 plus magnesium

The review^25^ included three small heterogeneous RCTs (33 participants) and pooling the data was not possible. One RCT did not report sufficient data to be analyzed. The second RCT did not find any significant differences between the treatment and placebo groups regarding social interactions, communication, compulsivity, impulsivity or hyperactivity. The last RCT focused on a subgroup of children with pervasive developmental disorders (PDDs) who exhibited clinical features similar to those with pyridoxine-dependent epilepsy. This small study (n = 8) only measured IQ and social quotient and found that there was a statistically significant benefit from vitamin B6 plus magnesium for IQ (MD = 5.2; 95% CI = 0.2 to 10.3).

The authors concluded that due to the small number of studies, the methodological quality of studies and the small sample sizes, no recommendations could be made regarding use of vitamin B6 plus magnesium for ASDs. For further details, the original abstract can be consulted, available from: http://onlinelibrary.wiley.com/doi/10.1002/14651858.CD003497.pub2/abstract.

## DISCUSSION

This review found that despite increasing prevalence of ASDs, there are still few systematic reviews and there is a scarcity of high-quality randomized trials addressing interventions for improving the main clinical features of ASDs. None of the reviews included here provided high-quality evidence for any outcome. Even more surprisingly, some of the interventions studied do not have any proven pathophysiological link with the disease or any well-known potential therapeutic mechanism (such as gluten and casein-free diets, omega-3 fatty acids and chelating agents). Furthermore, many of the off-label interventions used present no benefit for patients and could also increase the risk of adverse events.

Overall, the primary studies from each of the systematic reviews included had limited methodological quality, small sample sizes, short-term measurements of outcomes and poor reporting of adverse events. All of these facts increase the uncertainty surrounding the effects of the pharmacological and non-pharmacological interventions that are frequently used in clinical practice as options for patients with ASDs.

Regarding the implications for practice, some non-pharmacological interventions seem to be useful, given that they present some beneficial effects with few adverse events (albeit based on very low to low-quality evidence). These include gluten and casein-free diets, acupuncture, early intensive behavioral intervention, music therapy, parent-mediated early intervention, social skill groups and the Theory of Mind cognitive model. Their use needs to be discussed with patients, parents and caregivers in order to clarify the uncertainties regarding the results, time taken and costs.

Among the pharmacological options, aripiprazole, risperidone tricyclic antidepressants (clomipramine and tianeptine) and selective serotonin reuptake inhibitors (this last category only for adults) seem to have some benefits for specific symptoms, but the adverse events that have been reported need to be carefully considered before making decisions.

Regarding the implications for further research, this review of reviews makes it clear that much needs to be done on the therapeutics of ASDs. Firstly, experimental research exploring physiopathological mechanisms needs to be conducted in order to support further clinical studies. Subsequently, good-quality long-term clinical trials are required.

## CONCLUSION 

This review included 17 Cochrane systematic reviews. None of them provided high-quality evidence for any autism-related outcome. Acupuncture, early intensive behavioral intervention, gluten and casein-free diets, music therapy, parent-mediated early intervention, social skill groups and the Theory of Mind cognitive model seem to have benefits for patients with autism spectrum disorders (very low to low-quality evidence). Aripiprazole, risperidone, clomipramine, tianeptine and selective serotonin reuptake inhibitors are pharmacological options that seem to have some benefits (this last one only for adults), but all of them are associated with high risks of important adverse events. Experimental research to confirm the links between therapeutic options and the disease is needed, followed by high-quality long-term clinical trials.
